# The relationship between appetite hormones and body mass index in children with intoxication type metabolic diseases

**DOI:** 10.1186/s13023-026-04375-6

**Published:** 2026-06-18

**Authors:** Nevra Koç, Dilara Berşan Konyalıgil, Burcu Özen Yeşil, Beyza Kaldırım, Kübra Tel Adıgüzel, Oya Kıreker Köylü, Çiğdem Seher Kasapkara, Esra Fırat Oğuz, Funda Eren, Merve Esgi, Özlem Ünal Uzun, Mehmet Gündüz

**Affiliations:** 1 Department of Nutrition and Dietetics, Gülhane Health Sciences Faculty, University of Health Sciences Turkey, Ankara, Türkiye; 2 Department of Nutrition and Dietetics, Faculty of Health Sciences, Atılım University, Ankara, Türkiye; 3 Department of Pediatric Metabolism, Ankara Bilkent City Hospital, Children Hospital, Ankara, Türkiye; 4 Department of Biochemistry, Ankara Bilkent City Hospital, Ankara, Türkiye; 5 Department of Pediatric Metabolism, Kocaeli University, Kocaeli, Türkiye; 6 Gazi University Faculty of Medicine, Department of Pediatric Nutrition and Metabolism, Ankara, Türkiye; 7 University of Health Sciences, Department of Pediatric Metabolism, Ankara Bilkent City Hospital, Children Hospital, Ankara, Türkiye

**Keywords:** Amino acid metabolism disorders, Appetite hormones, Body mass index, Low-protein diet therapy

## Abstract

**Background:**

The aim of this study is to comparatively examine the effects of appetite loss and insufficient energy intake, which trigger poor metabolic control parameters, on appetite hormones and body mass index in children with intoxication-type metabolic diseases, including Phenylketonuria, Organic Acidemia, Maple Syrup Urine Disease, and Urea Cycle Disorder, in comparison with a healthy control group.

**Methods:**

The study sample consisted of 40 healthy children and 43 children with intoxication-type metabolic diseases, aged between 1 and 18 years. The mean age of the participants was 7.5 ± 4.60 years. The study was conducted with a total of 83 children, including 39 girls (47.0%) and 44 boys (53.0%).

**Results:**

When the body mass index z-score values of all participants were examined, it was found that 2.6% had a body mass index below − 2 SD, 71.1% were within the range of (-2 SD to + 1 SD), and 25.3% were above + 1 SD. Peptide YY and adiponectin hormone levels were found to be significantly higher in the patient group compared to the control group (*p* < 0,05). However, no significant difference was observed between the groups in terms of ghrelin, insulin, and leptin hormone levels (*p* > 0,05). It was found that ghrelin hormone levels in the patient group significantly varied according to body mass index z-score distribution (*p* < 0,05). Children in the ≤-2 SD group had significantly higher ghrelin hormone levels compared to those in the (-2 SD to + 1 SD) and > + 1 SD groups (*p* < 0,05).

**Conclusions:**

Significant alterations occur in the levels and signaling of appetite hormones in amino acid metabolism disorders. These changes result from factors such as low-protein diets, toxic metabolite accumulation, and organ dysfunction. Appetite hormone dysfunction can negatively affect patients’ energy intake and overall metabolic balance, leading to a decline in quality of life and adherence to treatment. Therefore, regular monitoring of appetite hormones and individualized dietary interventions should be considered essential strategies in the long-term nutritional management of these patients.

## Background

Nutrition is the utilisation of nutrients required for growth, development, healthy living and physical activity in the organism by metabolic or biochemical means [1]. The utilisation of nutrients in the organism is not a simple event but a series of complex events catalysed by enzymes [2]. Some of the diseases that result from the absence or deficiency of enzymes due to genetic defects can be treated by eliminating unmetabolised nutrients from the diet or limiting their amount [3].

Metabolic diseases are pathological conditions that develop as a result of events related to the synthesis or catabolism of proteins, carbohydrates and fatty acids [4]. The general incidence of inherited metabolic diseases is 1:4000-1:5000. The incidence varies according to ethnicity, diagnostic tests used, newborn screening programmes, technological developments, reporting rates, awareness and, most importantly, the prevalence of consanguineous marriages in the community. These diseases, which show autosomal recessive inheritance, are relatively common in our country, where consanguineous marriages are frequent, with rates reported to be approximately 25% in the general population and up to 42% in some geographical regions. Although each of these diseases is rare when considered individually, together with the newly defined diseases they form an important group of diseases [4–6].

In intoxication-type metabolic diseases, the metabolic pathway is blocked at the level of enzymes or cofactors. Substances that accumulate in the steps prior to the metabolic block cause damage to various organs and systems. Amino acid metabolism disorders (maple syrup urine disease, tyrosinemia type I, phenylketonuria, non-ketotic hyperglycinemia), organic acidemias (propionic acidemia, methylmalonic acidemia, isovaleric acidemia, glutaric aciduria type 1), urea cycle disorders (citrullinemia type 1, argininemia), ornithine transcarbamylase deficiency, carbamoyl phosphate synthetase 1 deficiency, N-acetyl glutamate synthase deficiency) and disorders related to carbohydrate metabolism (galactosemia) are metabolic disorders included in the intoxication type subheading [7].

Diseases of amino acid metabolism lead to an accumulation of amino acids or their metabolites in the body. This accumulation causes toxic effects and damage at the cellular level. In these diseases, which are treated with a lifelong diet to prevent accumulation and reduce the symptoms of the disease, natural protein is restricted and 80% of dietary protein is provided by synthetic protein equivalents. Studies have reported that the amount and type of dietary protein intake affects appetite status and body weight regulation in normally fed individuals. In intoxication-type metabolic disorders, where synthetic proteins are used intensively, the effects of these proteins on appetite are not well understood [8, 9].

Disorders of amino acid metabolism can lead to changes in appetite hormone levels and signalling. These changes can occur as a result of factors such as low protein diets, accumulation of toxic metabolites and organ dysfunction. Appetite hormone dysfunction can adversely affect quality of life and treatment compliance by affecting energy intake and overall metabolic balance in patients [10]. Therefore, regular monitoring of appetite hormones and individualisation of dietary interventions may be considered an important strategy in the long-term management of patients. Affection of appetite hormones in diseases of amino acid metabolism is associated not only with dietary restriction, but also with metabolic stress, toxic metabolites and changes in organ function [11]. These changes in appetite regulation can affect patients’ quality of life and make it difficult to adhere to treatment. Monitoring of hormone levels and supportive therapies in treatment protocols can help to improve metabolic balance and appetite control.

The aim of this study was to investigate the effect of poor metabolic control parameters induced by loss of appetite and inadequate energy intake on appetite hormones and body mass index (BMI) in children with phenylketonuria (PKU), organic acidemia (OA), maple syrup urine disease (MSUD) and urea cycle disorder (UCD), which are intoxication-type metabolic diseases, in comparison with a healthy control group. At the same time, the relationships between appetite hormones and BMI in children with PKU/OA/MSUD/UCD treated with a disease-specific diet will be determined.

## Methods

### Sample

The study was initiated following the approval of the Clinical Research Ethics Committee of Gülhane Health Sciences University Training and Research Hospital with decision number 2022/9 dated 02/03/2022.

The project was considered worthy of support by TÜBİTAK ARDEB with decision letter dated 20/05/2023 and project number 123S520.

This research was carried out between February 2022 and November 2024 at Ankara Bilkent City Hospital, Children’s Hospital, Department of Paediatrics, Division of Metabolism between February 2022 and November 2024 with PKU/OA/MSUD/UCD patients aged 1–18 years and a healthy control group of the same age and sex. During the execution of the project, the project was stopped for 5 months in order to reach the sufficient sample number and the project was approved to support the patient and sample group of Kocaeli University Faculty of Medicine, Internal Medicine, Division of Paediatric Metabolism from 05.04.2023.

Minitab 17.1.0 was used to calculate sample size. When the minimum number of samples required to be included in the study was selected as 80 for the 95% 4-level factor, a power analysis was performed by estimating a maximum difference of 0.06 between the means, and the power was found to be 90%.

The study sample consisted of 43 patients aged 1–18 years and 40 healthy children who agreed to participate in the study. Children who were outside this age range and who had an additional metabolic disorder were not included in the study. In the control group, obese subjects were excluded from the study.

The age and sex group to be included in the study was selected by taking into consideration the individuals who applied to the Paediatric Metabolism Department of Ankara Bilkent City Hospital, Kocaeli University Faculty of Medicine, Internal Medicine Department, Paediatric Metabolism Polyclinics and Diet Polyclinics. All patients followed by the team were included in the study due to the small number of patients with rare metabolic diseases.

**Inclusion criteria for the patient group;**

- Patients admitted to the Metabolism Outpatient Clinic for PKU/OA/MSUD/UCD.
- Between the ages of 1–18
- Family volunteering
- Parent or child is communicative and has adequate language skills

**Inclusion criteria for the control group;**

- Healthy children of the same age and sex who applied for nutritional counselling at the Diet Polyclinic.
- Family volunteering
- The parent or child is communicable and has adequate language skills.

**Exclusion criteria for the patient group;**

- Infant patients (0–1 year)
- Any additional disease diagnosed by a physician and/or any disease causing appetite disturbance.
- Non-voluntary behaviour of the family
- Parent or child is not communicative, has insufficient language skills

**Exclusion criteria for the control group;**

- Infants (0–1 years old)
- Presence of any chronic disease in the child
- Non-voluntary behaviour of the family
- Parent or child is not communicative, has insufficient language skills
- The child is obese
- The child is overweight or underweight.

The diagnosis of PKU and MSUD was established using plasma amino acid analysis and targeted gene panel testing. The diagnosis of OA and UCD was based on plasma amino acid analysis, carnitine and acylcarnitine profile, urine organic acid analysis and targeted gene panel testing. Metabolic stability during treatment was defined according to disease-specific biochemical parameters and clinical follow-up, including the absence of acute metabolic decompensation requiring hospitalization.

Families and children were given preliminary information about the study by the researcher. The informed consent form was signed by mothers and children who met the inclusion criteria and volunteered to participate in the study.

### Appetite status visual analogue scale

A questionnaire containing questions about dietary habits and appetite status (Visual Analogue Scale (VAS)) and sensory evaluation of disease-specific formulas used by healthy children aged 1–18 years in the same age and sex group who were diagnosed with PKU/OA/MSUD/UCD at the outpatient clinic was administered to the parent or child. The questionnaire was administered in a face-to-face interview between the researcher and the parent or child bringing the child.

### **Appetite hormones**

Venous blood samples were collected from the participants between 8:00 a.m. and 10:00 a.m. after an 8–12 h fasting into vacutainer tubes and centrifuged at 1300 × g for 10 min. Separated sera were aliquoted into eppendorf tubes and stored at − 80 °C until analysis. Human Peptide YY, Human Leptin, Human Adiponectin/Acrp30 and Human Insulin levels were measured with commercially available ELISA kit (Elabscience, Texas, USA) using the quantitative sandwich enzyme immunoassay technique. The optical density (OD) is measured spectrophotometrically using a microplate reader at a wavelength of 450 nm. The OD value is proportional to the concentration of human Peptide YY, Leptin, Adiponectin and Insulin levels. The concentration of the samples were calculated by comparing the OD of the samples to the standard curve. The detection range of the Peptide YY assay (catalog no: E-EL-H1237-lot no: FU1140HP6526) was 31.25–2000 pg/mL and the sensitivity of the assay was 18.75 pg/mL. Intra- and interassay precision were < 10% and < 10%, consecutively. The detection range of the Leptin assay (catalog no: E-EL-H6017-lot no: FU158R266529) was 15.63–1000 pg/mL and the sensitivity of the assay was 9.38 pg/mL. Intra- and interassay precision were < 10% and < 10%, consecutively. The detection range of the Adiponectin assay (catalog no: E-EL-H6122-lot no: FU1622ZN9655) was 0.16-10 ng/mL and the sensitivity of the assay was 0.1 ng/mL. Intra- and interassay precision were < 10% and < 10%, consecutively. The detection range of the Insulin assay (catalog no: E-EL-H2665-lot no: FU1262JJ9652) was 0.78-50 µIU/mL and the sensitivity of the assay was 0.47 µIU/mL. Intra- and interassay precision were < 10% and < 10%, consecutively.

Human Ghrelin were measured with commercially available ELISA kit (Elabscience, Texas, USA) using the competitive sandwich enzyme immunoassay technique. The concentration of the samples were calculated by comparing the OD of the samples to the standard curve. The detection range of the Ghrelin assay (catalog no: E-EL-H1919-lot no: FU14TFHB3090) was 0.16-10 ng/mL and the sensitivity of the assay was 0.1 ng/mL. Intra- and interassay precision were < 10% and < 10%, consecutively.

### Study design

When the child came to the outpatient clinic, a routine examination was carried out by the doctor and then the child was referred to the researcher who would collect the data if the criteria were met. Questionnaire; Physical examination of the child (weight, height, BMI, weight standard deviation score (SDS), height SDS, BMI SDS), clinical diagnosis, heel prick blood screening results, biochemical values at the time of diagnosis, use of additional treatment medication, use of special amino acid mixture, amino acid mixture used, amount consumed, number of meals, method of consumption pattern of amino acid mixture, appetite status (VAS (a number between 1 and 10 was selected)), questions on sensory evaluation of amino acid mixtures (Likert scale (a number between 1 and 5 was selected)), laboratory data (ghrelin (acylated ghrelin), insulin, leptin, Peptide YY, adiponectin (stoichin) levels), metabolic control parameters of the paediatric metabolic outpatient clinic (plasma amino acid levels, blood gases, ammonia levels used for the diagnosis and monitoring of metabolic disorders; written separately for each disease), medical nutrition therapy protocol (total amount of energy, protein and amino acid intake for each disease was questioned) and 3-day detailed food consumption record. The percentage of energy derived from protein was calculated as (protein intake (g) × 4 / total daily energy intake (kcal)) × 100. Heel prick blood screening results obtained from the National Newborn Screening Program, which screens newborns using tandem mass spectrometry (LC/MS/MS), were recorded.

Among the anthropometric measurements, height was measured in the recumbent position with an infantometer until the age of two years and with a stadiometer after the age of two years. Body weight was measured using a digital scale. SDS values were calculated for weight, height and BMI. Routine biochemical parameters such as blood/urine were obtained from patient records.

The research team physicians performed the examinations in the outpatient clinic and ensured that the routine examinations were performed, the questionnaires were administered, and the data were transferred to the statistical environment. All members of the research team ensured that the data were analysed and that the analysis was written up in an article.

### Statistical analysis of data

Statistical analyses of data obtained from questionnaires and measurements were performed using IBM SPSS Statistics version 21.0 in a Windows environment [12]. The mean, standard deviation, median, mode and mode were calculated for the data. The distribution of qualitative data was presented in tables of numbers and percentages. For comparison of binary groups, Independent T test was used for those showing normal distribution in two independent groups, one-way ANOVA test was used for those showing normal distribution in more than two independent groups, Mann-Whitney U test was used to compare the difference between those not showing normal distribution in two independent groups. Multiple linear regression analysis was used to examine the relationship between parameters. Food consumption records were analysed using the Computer Assisted Nutrition Programme Nutrition Information System 8.1 (BEBIS). Results were considered significant if *p* < 0.05 with a 95% confidence interval.

## Results

### General characteristics of the participants

The study sample consisted of 40 healthy children and 43 children with intoxication-type metabolic disorders. Table 1 shows the age, sex, weight, height and BMI of the children. The mean age of the children participating in the study was 7.5 ± 4.60 years. A total of 83 children were included in the study, 39 girls (47.0%) and 44 boys (53.0%). The mean body weight, height and BMI of the children were 29.2 ± 19.62 kg, 122.2 ± 29.62 cm and 17.4 ± 4.03 kg/m2, respectively. When the BMI z-score values of the children participating in the study were analysed, it was found that 2.6% of the children had a BMI below − 2 SD, 71.1% had a BMI between − 2 SD and + 1 SD, and 25.3% had a BMI above + 1 SD. A comparison of BMI SDS between the disease group and the control group showed no statistically significant difference (*p* > 0.05), indicating that the groups were comparable in terms of BMI.

### Characteristics of the disease

The characteristics of the children with intoxication-type metabolic disorders were evaluated in relation to their disease status. The clinical diagnosis was PKU in 16.7% (n:14) of the children, UCD in 13.1% (n:11), MSUD in 11.9% (n:10) and OA in 9.5% (n:8). 44.2% (n:19) of the children were taking medication, 86% (n:37) were using low protein products and 86% (n:37) were using special amino acid mixtures. Of the children using amino acid blends, 23.3% (n:10) took the amino acid blend with another food.

### Assessment of appetite status in patients and controls

When the appetite status of the children participating in the study and the sensory evaluation of the amino acid mixtures in the children with intoxication-type metabolic disorders were asked, it was found that the appetite evaluation of the children was 5.6 ± 2.60 on average between 1 and 10 values, while this value was 6.3 ± 1.81 in the control group and the difference between the groups was not statistically significant (*p* > 0.05). When the sensory evaluation of the amino acid mixture was asked in the sick children, it was observed that the taste was evaluated with 2.3 ± 1.42, the smell with 2.2 ± 1.52 and the appearance with 3.8 ± 1.18 between 1 and 5 values (Table 1).

**Table 1:** Sensory evaluation of amino acid mixture and appetite status in children with intoxication-type metabolic disorders

Variable	Patient Group $\bar{\mathrm{X}}$±SD	Control Group $\bar{\mathrm{X}}$±SD	*p*
How do you rate appetite?	5.6 ± 2.60	6.3 ± 1.81	0.174^a^

Variable	$\bar{\mathrm{X}}$±SD	(Lower-Upper)	
How would you rate the taste of the amino acid mixture consumed?	2.3 ± 1.42	(1–5)	
How would you rate the smell of the amino acid mixture consumed?	2.2 ± 1.52	(1–5)	
How would you rate the appearance of the amino acid mixture consumed?	3.8 ± 1.18	(1–5)	

^a^The Independent Samples t Test, *p* < 0,05

### Biochemical test results of children with intoxication-type metabolic disorders

The evaluation of the disease-specific blood findings of the children with intoxication-type metabolic disorders who participated in the study is shown in Table 2.

**Table 2:** Biochemical tests

Clinical Diagnosis	Blood Findings	Result
PKU	Phenylalanine	371.6 ± 199.96 µmol/L
OA	Bicarbonate Carbon dioxide Blood glucose pH Ketone bodies Ammonia Glycine	18.9 ± 3.53 mmol/L 30.2 ± 6.33 mmHg 92.0 ± 14.91 mg/dL 7.4 ± 0.04 0.8 ± 1.82 mmol/mol Krea 57.7 ± 45.86 µmol/L 648.5 ± 332.94 µmol/L
MSUD	Ketone bodies Blood glucose Leucine Isoleucine Valin	0.1 ± 0.51 mmol/mol Krea 110.2 ± 16.34 mg/dL 225.0 ± 44.90 µmol/L 209.2 ± 84.04 µmol/L 141.9 ± 101.93 µmol/L
UCD	Ammonia Glutamine Alanine Tandem organic acid analysis	89.9 ± 124.68 µmol/L 484.8 ± 451.54 µmol/L 342.9 ± 362.08 µmol/L 856.7 ± 1166.35

PKU: Phenylketonuria, OA: Organic acidemia, MSUD: Maple syrup urinary disease UCD: Urea cycle defect

### Evaluation of appetite hormone levels according to diagnostic groups

The evaluation of the appetite hormone levels of the children with intoxication-type metabolic disorders who participated in the study according to the diagnostic groups is shown in Table 3.

**Table 3:** Evaluation of appetite hormone levels in children with intoxication-type metabolic disorders according to diagnostic groups

	Ghrelin (ng/mL) $\bar{\mathrm{X}}$±SD (Lower-Upper)	Insulin (uIU/mL) $\bar{\mathrm{X}}$±SD (Lower-Upper)	Leptin (pg/mL) $\bar{\mathrm{X}}$±SD (Lower-Upper)	Peptide YY (pg/mL) $\bar{\mathrm{X}}$±SD (Lower-Upper)	Adiponectin (ng/mL) $\bar{\mathrm{X}}$±SD (Lower-Upper)
PKU	1.7 ± 0.83 (0.70–3.37)	2.6 ± 2.80 (1.57–11.95)	2442.9 ± 4087.89 (479.46-15668.83)	230.1 ± 70.25 (135.26-341.34)	52302.2 ± 17918 (2370.97-68801.53)
OA	2.2 ± 1.53 (0.50–4.36)	3.0 ± 2.65 (1.41–8.40)	2096.8 ± 4845.83 (528.63-13846.64)	194.2 ± 42.38 (145.31-263.56)	51865.3 ± 21884.31 (975.77-67740.91)
MSUD	1.6 ± 1.32 (0.37–4.02)	7.6 ± 8.40 (1.06–28.84)	2111.7 ± 1808.19 (457.40-5244.28)	170.3 ± 42.42 (134.29-285.93)	58121.1 ± 14644.90 (19061.54-68176.16)
UCD	1.7 ± 0.78 (0.52–2.83)	4.9 ± 7.05 (1.54–25.93)	1584.8 ± 726.86 (733.48-2961.87)	224.4 ± 173.40 (140.97-733.53)	59500.4 ± 13865.12 (23349.58-71968.58)
Control	1.4 ± 0.88 (0.16–3.86)	4.3 ± 3.93 (1.08–16.77)	2259.6 ± 2647.43 (481.96-9063.13)	167.2 ± 56.24 (134.20-424.22)	50443.4 ± 16299.27 (590.33-69555.14)

PKU: Phenylketonuria, OA: Organic acidemia, MSUD: Maple syrup urinary disease UCD: Urea cycle defect

The comparison of appetite hormone levels between patients and controls is shown in Table 4. Peptide YY and adiponectin hormones were found to be higher in the patient group compared to the control group and this difference was statistically significant (*p* < 0.05). Ghrelin, insulin and leptin hormones showed no significant difference between the groups (*p* > 0.05).

**Table 4:** Comparison of appetite hormone levels in children participating in the study

Appetite hormones	Patient Group $\bar{\mathrm{X}}$±SD	Control Group $\bar{\mathrm{X}}$±SD	*P*
Ghrelin	1.8 ± 1.08	1.4 ± 0.88	0.107^a^
Insulin	2.8(1.0–28.8)	2.5(1.0-16.7)	0.884^b^
Leptin	1065.3(457.4 -15668.8)	829.4(481.9–9063.1)	0.613^b^
Peptide YY	166.3(134.2–733.5)	145.8(134.2-424.2)	**0.002** ^**b**^
Adiponectin	60487.6(958.7–71968.5)	54031.5(590.3-69555.1)	**0.015** ^**b**^

^a^Independent two sample t test ± S, ^b^Mann Whitney U test [Median (min-max)], *p* < 0,05

### Multiple regression analysis of the relationship between the smell of amino acid mixtures and appetite hormones

In Table 5, the effect of the smell of amino acid mixtures consumed by children with intoxication-type metabolic disorders on appetite hormones was evaluated by multiple regression analysis. No significant relationship was observed between the rating of the smell of the amino acid mixture and appetite hormones (*p* > 0.05).

**Table 5:** Multiple linear regression analysis of the relationship between the odor of consumed amino acid mixtures and appetite hormones

Variable	B	*p*	t	%95 CI
Ghrelin	-0.318	0.250	-1.172	-0.871, 0.235
Insulin	-0.045	0.303	-1.048	-0.133, 0.043
Leptin	4.187	0.961	0.049	0.000, 0.000
Peptide YY	-0.002	0.434	-0.793	-0.007, 0.003
Adiponectin	-9.604	0.551	-0.603	0.000, 0.000

B: regression coefficient; CI: confidence interval

Multiple linear regression analysis; *p* < 0,05

### ANOVA results for appetite hormones according to BMI values z Score Classification

When analysing Table 6, it was observed that the ghrelin hormone values of the children with intoxication-type metabolic disorders differed significantly according to the BMI z-score distribution (F = 6.522) (*p* < 0.05). The Hochberg test was used to determine between which BMI groups the ghrelin hormone levels of the children with intoxication-type metabolic disorders differed. According to the test results, it was observed that the ghrelin hormone value of children in the ≤-2SD group (X̄=3.5) was significantly higher than that of the (-2SD - +1SD) (X̄=1.8) and > + 1SD (X̄=1.3) groups according to the BMI z-score distribution (*p* < 0.05). When insulin, leptin, peptide YY and adiponectin hormones were analysed, no significant difference was observed according to the BMI z score distribution (*p* > 0.05).

**Table 6:** ANOVA results of appetite hormones of children according to BMI z-score classification

Variable	Groups	*N*	$\bar{\mathrm{X}}$±SD	F	df	*P*	Difference
Ghrelin	**≤-2SD** ^**1**^	3	3.5 ± 1.12	6.522	2 40	0.004*	**1 > 2**,**3**
	**-2SD - +1SD** ^**2**^	30	1.8 ± 1.01	6.522	2 40		
	**>+1SD** ^**3**^	10	1.3 ± 0.69	6.522	2 40		
Insulin*	**≤-2SD** ^**1**^	3	9.7 ± 14.02	1.254	2 5.028	0.728	
	**-2SD - +1SD** ^**2**^	30	4.6 ± 5.39	1.254	2 5.028		
	**>+1SD** ^**3**^	10	3.81 ± 3.18	1.254	2 5.028		
Leptin	**≤-2SD** ^**1**^	3	805.5 ± 446.78	2.076	2 40	0.139	
	**-2SD - +1SD** ^**2**^	30	1850.7 ± 2899.05	2.076	2 40		
	**>+1SD** ^**3**^	10	3958.6 ± 3190.8	2.076	2 40		
Peptide YY	**≤-2SD** ^**1**^	3	194.8 ± 7.86	0.055	2 40	0.947	
	**-2SD - +1SD** ^**2**^	30	202.5 ± 114.40	0.055	2 40		
	**>+1SD** ^**3**^	10	190.7 ± 56.32	0.055	2 40		
Adiponectin*	**≤-2SD** ^**1**^	3	45112.0 ± 38513.95	0.737	2 4.546	0.825	
	**-2SD - +1SD** ^**2**^	30	57002.6 ± 11507.82	0.737	2 4.546		
	**>+1SD** ^**3**^	10	53745.4 ± 22714.20	0.737	2 4.546		

*Variance between groups is not homogeneously distributed. Welch test considered, *p* < 0.05

The mean daily energy consumption of the children with intoxication-type metabolic disorders was 2012.5 ± 999.69 kcal, while that of the control group was 1505.2 ± 454.75 kcal, and the difference between the groups was statistically significant (*p* < 0.05). While no statistically significant difference was observed between the mean daily protein consumption of the children (*p* > 0.05), the mean daily amino acid consumption of the patient group was lower in all amino acid groups compared to the control group and this difference was statistically significant (*p* < 0.05) (Table 7).

**Table 7:** Average daily energy and nutrient intakes of children in the study

	Patient Group (*n*:43)	Control Group (*n*:40)	*P*
Energy (kcal)	2012.5 ± 999.69	1505.2 ± 454.75	**0.004** ^**a**^
Water (g)	915.4 ± 431.92	1395.5 ± 541.29	**0.000** ^**a**^
Alcohol (g)	0.0(0.0-0.1)	0.0(0.0-0.5)	0.624^b^
Protein (g)	47.5(8.7-377.1)	50.9(9.1-101.7)	0.266^b^
Protein (%)	16.0(4.0–51.0)	13.0(8.0–22.0)	0.223^b^
Fat (g)	75.8 ± 40.62	61.6 ± 22.17	0.051^a^
Fat (%)	35.0 ± 11.05	36.6 ± 6.80	0.425^a^
PUFAs (g)	11.4 ± 6.93	11.7 ± 6.58	0.843^a^
Cholesterol (mg)	110.9 ± 141.18	293.4 ± 201.67	**0.000** ^**a**^
CHO (g)	219.6 ± 107.47	184.8 ± 65.15	0.076^a^
CHO (%)	46.7 ± 11.26	49.5 ± 7.60	0.185^a^
Dietary fibre (g)	13.9(2.7–34.1)	13.6(2.1–45.9)	0.966^b^
Vitamin A (µg)	1559.2(268.0-19371.1)	1030.0(390.2-2052.2)	**0.001** ^**b**^
Carotene (mg)	5.4(0.0-115.5)	2.2(0.3-221.7)	**0.004** ^**b**^
Vitamin E (mg)	19.4(2.9–176.0)	10.1(1.7–34.0)	**0.006** ^**b**^
Thiamine (mg)	1.5(0.1–21.7)	0.7(0.1–1.9)	**0.024** ^**b**^
Riboflavin(mg)	1.8(0.2–23.8)	1.2(0.2–2.5)	0.095^b^
Vitamin B6 (mg)	1.5(0.3–20.1)	1.0(0.1–2.3)	**0.007** ^**b**^
Folate (µg)	262.8(17.4-2346.8)	251.9(29.4-612.8)	0.292^b^
Vitamin C (mg)	172.7(30.3-1021.1)	89.2(16.2-270.7)	**0.000** ^**b**^
Sodium (mg)	2830.7 ± 1702.46	2331.6 ± 1039.65	0.109^a^
Potassium (mg)	2670.5(828.4-13234.6)	2074.7(544.3-4000.4)	**0.046** ^**b**^
Calcium (mg)	1397.3(169.6-22086.8)	645.5(183.5-1453.3)	**0.008** ^**b**^
Magnesium (mg)	269.4(72.9–3074.0)	206.8(52.8–477.0)	**0.012** ^**b**^
Phosphorus (mg)	1185.6(146.2-13197.8)	894.4(162.6–1916.0)	0.149^b^
Iron (mg)	20.8(1.3-266.4)	7.0(1.2–20.8)	**0.000** ^**b**^
Zinc (mg)	16.7(1.9-190.3)	6.7(1.1–19.0)	**0.006** ^**b**^
Tryptophan (mg)	176.8 ± 200.40	509.4 ± 292.40	**0.000** ^**a**^
Non-essential aa (g)	7.7 ± 9.07	21.6 ± 12.11	**0.000** ^**a**^
Serine (mg)	720.8 ± 857.64	2281.9 ± 1300.29	**0.000** ^**a**^
Glutamic asid (mg)	3259.4 ± 3920.51	8956.1 ± 4837.45	**0.000** ^**a**^
Proline (mg)	1059.6 ± 1329.14	3384.9 ± 1898.41	**0.000** ^**a**^
Aspartame (mg)	1417.8 ± 1421.56	3677.9 ± 2080.64	**0.000** ^**a**^
Essential aa (g)	6.6 ± 8.63	21.2 ± 12.73	**0.000** ^**a**^
Histidine (mg)	329.9 ± 260.46	1040.0 ± 662.76	**0.000** ^**a**^
Arginine (mg)	766.5 ± 975.86	2109.9 ± 1289.09	**0.000** ^**a**^
Valine (mg)	75.,2 ± 916.29	2464.2 ± 1437.58	**0.000** ^**a**^
Threonine (mg)	545.0 ± 688.22	1713.3 ± 1033.37	**0.000** ^**a**^
Tyrosine (mg)	488.1 ± 604.27	1605.4 ± 950.76	**0.000** ^**a**^
Phenylalanine (mg)	660.4 ± 793.66	2004.7 ± 1128.98	**0.000** ^**a**^
Cystine (mg)	213.8 ± 273.58	563.2 ± 301.45	**0.000** ^**a**^
Methionine (mg)	286.8 ± 390.60	937.4 ± 573.62	**0.000** ^**a**^
Isoleucine (mg)	563.0 ± 625.83	2163.6 ± 1291.42	**0.000** ^**a**^
Lysine (mg)	657.4 ± 912.64	2692.8 ± 1782.53	**0.000** ^**a**^
Leucine (mg)	941.2 ± 1008.31	3498.5 ± 2045.29	**0.000** ^**a**^
Alanine (mg)	437.5(24.3-4569.7)	1540.4(326.3-5476.8)	**0**,**000**^**b**^

^a^Independent two sample t test ± S, ^b^Mann Whitney U test [Median (min-max)], *p* < 0,05, CHO: Carbohydrate, PUFAs: Polyunsaturated fatty acids AA: Amino acid

## Discussion

This study was conducted to investigate the effect of poor metabolic control parameters induced by loss of appetite and inadequate energy intake on appetite hormones and BMI in 43 children aged 1–18 years with PKU, OA, MSUD and UCD, which are intoxication-type metabolic disorders, and to evaluate the results obtained in this study by comparing them with a control group of 40 healthy children.

Ghrelin, one of the appetite hormones, is a hormone involved in the regulation of body weight, appetite and eating behaviour [13]. In the study, no significant difference was observed between the ghrelin hormone and the patient and control groups (*p* > 0.05), but a significant difference was observed between the ghrelin homone values and the BMI z-score distribution in children with intoxication-type metabolic disease (*p* < 0.05). It was found that ghrelin hormone levels were significantly higher in patients with BMI z-score below − 2 SD (*p* < 0.05).

It is known that plasma ghrelin concentrations decrease in obesity and increase with weight loss. In a review of the literature, the only studies of the hormone ghrelin in metabolic disorders of the intoxication type found were in patients with PKU. Similar to this study, a study in children with poor metabolic control showed that fasting plasma ghrelin concentrations decreased [10]. Another study reported that low fasting plasma ghrelin concentrations in children with poor metabolic control were responsible for the prevalence of obesity in children with PKU [14].

Leptin is a hormone synthesised and secreted by fat cells that suppresses food intake and increases energy expenditure by acting on the hypothalamus. In this study, no significant difference was observed between leptin hormone and the patient and control groups, or between leptin hormone and BMI levels (*p* > 0.05). In a previous study, in contrast to this study, elevated plasma phenylalanine concentration, which is an indicator of poor metabolic control in patients with PKU, was associated with elevated leptin concentration [15]. In other intoxication-type metabolic disorders, no study was found that related to the hormone leptin.

The hormone insulin is secreted by the beta cells of the pancreas; it lowers blood sugar levels and inhibits the secretion of glucagon. It is one of the major anabolic hormones that regulate tissue energy use [16]. In this study, no significant difference was observed between insulin hormone and patient and control groups, and between insulin hormone and BMI levels (*p* > 0.05). No study was found in the literature between insulin hormone concentration and intoxication-type metabolic diseases.

Adiponectin hormone is secreted from adipose tissue and reduces hepatic glucose production and blood glucose levels [17]. In this study, adiponectin hormone was found to be significantly higher in the patient group than in the control group (*p* < 0.05). In a review of the literature, it has been reported that plasma adiponectin levels were higher in patients with PKU compared to the control group in a study that included patients with non-compliant diet and a healthy control group [18]. There are no studies on the hormone adiponectin in other intoxication-type metabolic disorders.

Peptide YY is a hormone involved in appetite suppression [19]. In this study, peptide YY hormone was found to be significantly higher in the patient group than in the control group (*p* < 0.05). In the literature, similar to the results of this study, there are studies showing that peptide YY hormone is high in intoxication-type metabolic disorders [8, 9, 16, 20–22].

The smell, taste and nutrient content of the food consumed are important factors in determining appetite preference [23]. When children with intoxication-type metabolic disorders were asked to rate the sensory quality of the amino acid mixture, they rated taste with a mean score of 2.3 ± 1.42 (‘disliked’), smell with a mean score of 2.2 ± 1.52 (‘disliked’) and appearance with a mean score of 3.8 ± 1.18 (‘liked’) on a scale of 1 to 5. No significant relationship was found between the smell of the amino acid mixtures and appetite hormones. According to a study in the literature, the effect of a food on appetite may be related to its taste, as indicated by its smell, rather than its macronutrient content [23].

## Conclusions

Appetite is an important factor controlled by the hypothalamus and initiates food intake. In this study, inadequate energy intake and dietary compliance were found to affect poor metabolic control parameters and appetite hormones in children with PKU/UCD/OA/MSUD.

In the dietary treatment of children with PKU/UCD/OA/MSUD, it was found that the sensory properties of synthetic amino acid mixtures given to meet protein requirements due to natural protein restriction in the diet affected appetite.

It was shown that inadequate energy intake, developed with poor metabolic control parameters and sensory properties of disease-specific amino acid mixtures, caused loss of appetite and affected BMI in children with PKU/UCD/OA/MSUD.

In intoxication-type metabolic disorders, there may be marked changes in the levels of appetite hormones. These changes can occur as a result of factors such as low protein diets, accumulation of toxic metabolites and organ dysfunction. Appetite hormone dysfunction can adversely affect quality of life and treatment compliance by affecting energy intake and overall metabolic balance. Therefore, in children with intoxication-type metabolic disorders, regular monitoring of appetite hormones, measures to improve the taste and sensory properties of amino acid mixtures and individualisation of diet/medical nutrition therapy should be considered as an important strategy in the long-term management of patients.

It is noted that there are no studies in the literature that have investigated intoxication-type metabolic disorders and appetite hormones. Although there are studies conducted in patients with PKU, this study was conducted as a comprehensive study including PKU/UCD/OA/MSUD.

Further product development studies are needed to extend this two-centre trial, to provide appetite control in the treatment of children with intoxication-type metabolic disorders, and to highlight the taste and appetite-enhancing properties of disease-specific amino acid blends that should be taken with a lifelong diet.

## Data Availability

The original data supporting the results of this study are available on reasonable request from the corresponding author.

## Abbreviations

BMI: Body mass index
PKU: Phenylketonuria
OA: Organic acidemia
MSUD: Maple syrup urine disease
UCD: Urea cycle disorder
VAS: Visual Analogue Scale
OD: Optical density
SDS: Standard deviation score
BEBIS: Computer Assisted Nutrition Programme Nutrition Information System 8.1
